# Water-Proof Boots

**Published:** 1869-07

**Authors:** 


### WATER-PROOF BOOTS.

If Petroleum is occasionally applied with a small brush, between the upper edge of the sole and the upper leather, it will exclude water, whether in sewed or pegged boots; while the latter are preserved and kept tighter. The entire upper leather may be made impervious to water, if you boil one quart linseed oil, with half a pound Venice turpentine, with which paint the leather frequently while warm, but not hot, till the leather will absorb no more.

### BORAX

Is one of our most useful and harmless drugs. It dissolves in twelve times its weight in cold, and twice its weight in hot water. It is a white salt. An ounce of it costs but a few cents, and a single teaspoonful dissolved in half a teacup of warm water and rubbed into the roots of the hair, is the best

### HAIR-WASH

That can be used, as it finds about the scalp and hair a little oil, with which it combines, and makes a mild soap, which soon lathers the whole head, and leaves the entire scalp and "every particular hair" perfectly clean and silken. It should be rubbed in patiently with the balls of the fingers; the latter should be washed off with an abundance of warm water, and the hair dried with soft towels. If used once every two or three weeks, it would keep the scalp entirely free from dandruff, which has such disgusting associations when seen on the shoulders of a man's coat; for what is

### DANDRUFF

But the dust and dirt which settles on the head, makes its way down to the roots of the hair, there combines with the natural oil of the parts, and the evaporations of the effete matters of the scalp make solid flakes of greasy dirt, which, drying, scale off, and fall down upon the coat collar. Two table-spoonfuls of soda, put into a tub of hot water, and soiled clothes being allowed to soak in it for several hours, would save one half the hard labor of

WASHING-DAY

By its capability of dissolving and absorbing the dirt from whatever fabrics are washed. If the kitchen floor is swept and then sprinkled with Borax, and is also scattered into the cracks and on the tables and shelves, in two or three evenings there will not be found a single live COCKROACH.

WORDS OF HOPE. 256 pp. 12mo. An admirable book for all Christian readers: God Chasteneth in Love; Trust in God; Fruit of Sorrow; Children in Heaven; Death; Eternal Home.

GLOVERSON and his Silent Partners, by Ralph Keeler. 372 pp. 12mo. Dedicated to the Hon. George P. Marsh, U. S. Ambassador to the Italian Court.

CHARLES BELL, the Waif of Elm Island, by Rev. Elijah Kellogg: a good book for boys who want to become men in the highest sense. 325 pp. 12mo.

HILLSBORO' FARMS. 423 pp. 12mo. By Sophia Dickinson Cobb.

DR. HOWELL'S FAMILY, by Mrs. H. B. Goodwin, author of "Madge," "Sherwood," &c. 361 pp. 12mo.

Among other interesting and valuable publications of this enterprising house are "From the Oak to the Olive," being a plain record of a Pleasant Journey, by Mrs. Julia Ward Howe. 423 pp. 12mo; $2.00. Henry Giles's "Human Life in Shakspeare." $2.00. "On Nurses and Nursing," and the Management of Sick Women, by Prof. H. R. Storer, M.D. $1.00. All the books named are in print, paper, binding and style worthy of the taste and judgment of the publishers, and would be ornaments to any library or centre table.

"MY PARIS," a 12mo of 336 pp., being FRENCH CHARACTER SKETCHES by Edward King, and published by Loring, 319 Washington St., Boston, Mass. Mr. King was the correspondent of the *Springfield Republican* and Boston *Transcript* during the Exposition year. The public appreciation of this correspondence was so unexpectedly generous, it was thought desirable that the embodiment in book form of what was written, with additional incorporations, would answer a good purpose. Mr. King was a stranger in Paris, and says he was a young man. Happy fellow! this prepares us for a vivacious volume, no gall, no vinegar; these come later, when writers become dyspeptic, rheumatic, and gouty. A further, or inner key to the nature of the book is found in an expression in the author's preface, "People interest me more than places," "The best houses sometimes have the most unattractive doors." Our own personal observation corroborates much that Mr. King has written. Those who have been to Paris will find their memories agreeably refreshed on many points; those who contemplate going, will have their desire intensified by the perusal of this very spicy and attractive volume.

"Ante-Natal Infanticide" destroying the life of the unborn child is a printed discourse by the Rev. E. Frank Home, of Terra Haute, Indiana. This is a timely, temperate, and needed publication. The subject is handled with great delicacy by the Rev. gentleman. The author deserves the thanks of the church universal for this essay, and has shown that he is a faithful watchman on the outposts.

How to Keep Well, is an item of knowledge of incalculably greater importance than the ability to recover health after it has been lost. A main object in starting this Journal was the wish to show the young, especially theological students and clergymen, how to avoid disease. We feel the importance of this more deeply than others, because persons are calling upon us from time to time who express themselves as being willing to "give the world," if they had it, if it could purchase restoration to the glorious good health which they once enjoyed. Many a parent has come with a breaking heart, willing to beggar themselves, if it would only restore a promising son or loving daughter to robustness and bodily vigor; and yet, a few years earlier, a little instruction, costing a dollar or two, might have easily prevented it all. But most parents will sooner give ten dollars towards purchasing trashy novels and more trashy newspaper stories, than one for a publication like this which seeks to instruct its readers how to maintain that health without which life has no sunshine.

Health, like religion, is but too often neglected until it is too late, but there are some practical suggestions which are of such general importance that it answers a good purpose sometimes to repeat them, in the nature of line upon line, and precept upon precept, here a little and there a little, and over and over again. The following articles merit universal attention, because they are applicable more or less directly to all classes, ages and conditions of mankind; they are part of a series of Health Tracts, 350 in number, and which bound in one octavo volume, are sent from this office, pp., for $2.50.

